# Psychiatric Comorbidities and Environmental Triggers in Patients with Chronic Daily Headache: A Lifestyle Study

**Published:** 2017-01

**Authors:** Fakhrudin Faizi, Abbas Tavallaee, Aboulfazl Rahimi, Masoud Saghafinia

**Affiliations:** 1 PhD Candidate in Pain Research and Management, Behavioral Sciences Research Center, Baqiyatallah University of Medical Sciences, Tehran, Iran.; 2 Associated Professor of Psychiatry, Behavioral Sciences Research Center, Baqiyatallah University of Medical Sciences, Tehran, Iran.; 3 PhD, Assistant Professor, Faculty of Nursing, Baqiyatallah University of Medical Sciences, Tehran, Iran.; 4 Associated Professor, Pain Fellowship at Trauma Research Center, Baqiyatallah University of Medical Sciences, Tehran, Iran.

**Keywords:** *Comorbidity*, *Headache*, *Life Style*, *Psychiatry*, *Trigger*

## Abstract

**Objective: **Patients with chronic daily headache (CDH) suffer from several significant psychiatric comorbidities and have unhealthy lifestyle. We aimed at studying psychiatric comorbidities, environmental triggers, lifestyle factors, and intensity of CDH in patients referred by the department of neurology from 2011 to 2014.

**Method:** Through medical and psychiatric interviews and using 0 to 10 visual analogue scale (VAS), we assessed patients with CDH, using a checklist, to elicit psychiatric comorbidities, intensity of CDH, environmental factors, and lifestyle derangement.

**Results:** We interviewed 413 (age 16-80 years, mean 40 +/- 14.0) out of 548 patients; 312 (75.5%) were married, and 282 (68.1%) were female. Environmental triggers (374, 90.6%) were the most common cause of CDH, while 214 (51.8%) had no compliance to recommended nutrition. Exercise avoidance (201, 48.7%) was the less prevalent lifestyle factor. Of the patients, 372 (90.1%) were stressed and 162 (39.2%) had obsessive-compulsive disorder (OCD), which were the most and less prevalent psychiatric comorbidities, respectively. Intensity of pain was moderate to severe (mean score = 7.1+/- 1.9), while females reported higher VAS scores (p<0.02). Patients with previous history of psychotherapy reported higher score of VAS (p<0.001). Those patients living with a person suffering from head pain reported more VAS score (p<0.003).

**Conclusion**: Notable psychiatric comorbidities were found in patients with CDH, many of which are modifiable such as environmental triggers and unhealthy lifestyle. In heavily populated cities, these factors may double the burden of the CDH by precipitating new or exacerbating previous psychiatric comorbidities. We, thus, suggest conducting more studies on this subject.

Several studies have suggested that any comprehensive headache management plan should handle various headache-related comorbidities to lower the burden of the existing medical condition (1). Patients with chronic daily headache (CDH) suffer from several significant psychiatric comorbidities (2). While the patients detest to label their headache with psychiatric terms (3), depression and anxiety have been reported as the most prevalent comorbidities of CDH (4). However, environmental triggers such as traffic, noise, and rapid changes in the weather and altitude may also result in headache (5, 6). Several studies have reported that the patients have also unhealthy lifestyle (7). Sleep disturbances (8), diet, and nutritional triggers are also common in these patients (9). To date, no studies have been published from IR Iran that focused on probable initiating or exacerbating causes of CDH. Increased psychiatric disorders and CDH among the inhabitants of large cities in IR Iran have been reported in the recent years (10-14). In this study, we investigated the lifestyle of CDH patients living in crowded cities, mostly in Tehran and Karaj, to detect factors related to CDH.

## Materials and Methods

In this cross sectional survey, we studied patients complaining of CDH (headache on ≥ 15 days/month for >3 months or equal to or more than 180 days/year) based on International Classification of Headache Disorders -3 beta (15) referred by neurologists at a multidisciplinary headache clinic at the Baqiyatallah Hospital located in Tehran, IR Iran.

All patients have been visited earlier and were reassessed during October 2011 to the end of 2014 using in depth psychiatric interviews to find out any psychiatric comorbidity, deviation from the healthy lifestyle, and impact of environmental factors.

At first, a psychiatric interview was carried out by a psychiatrist (A.T.) to differentiate probable psychiatric disorders or comorbidities; patients suspected of having physical pain were reassessed by a pain medicine fellow (M.S.) to rule out any significant organic problems. The participants also agreed to characterize their lifestyle in detail with a checklist by a help of another research member (F.F.) Physical activity was outlined based on the American Heart Association (16) as nutritional triggers (17, 18) stress, and environmental factors (19). The Persian checklist was derived from the above-mentioned sources and its face and content validity were approved by both the research team and Behavioral Sciences Research Center (BSRC) of the Baqiyatallah University of Medical Sciences (BUMS) as the stakeholder and government supervisor. An 11-point (0-10) visual analog scale (VAS) (15) was used to measure the severity of the headache. The scale has previously been employed to assess the severity of chronic daily headache in the country as a reliable and valid tool (20). Inclusion criteria were experiencing headache for at least last 6 months and filling a written informed consent form. Having any major psychiatric problems, malingering, or refusal were defined as exclusion criteria. Patients were followed- up and data completion was carried out through phone calls. Data were analyzed using SPSS software for Windows, Version 18 (SPSS Inc. Chicago, USA, 2009). A value of probability (p value) less than 0.05 was assumed as significance level; and Mann-Whitney U, Chi-Square, and Fisher’s exact test were used for data analysis. Informed consent was obtained from the study participants and ethical approval of the work was obtained from Research Ethics Committee of the BSRC.

## Results

**Figure1:**
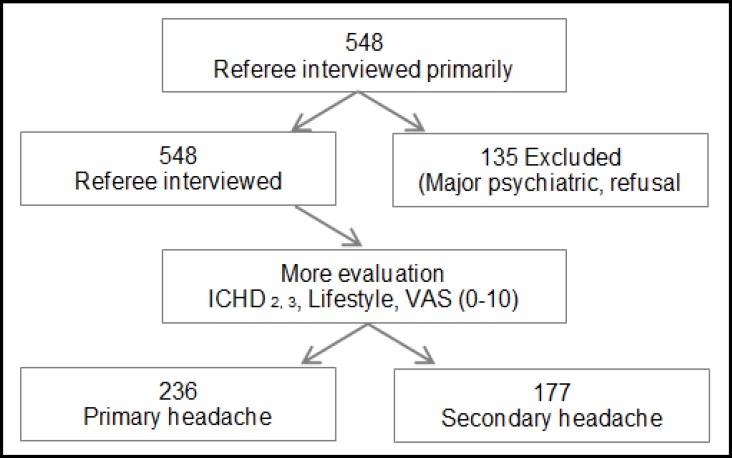
Flowchart of The Study: screening of Chronic Daily Headache

548 patients who were referred to the clinic were interviewed and 135 were excluded due to the presence of coexisting major psychiatric disorders, considerable organic problems, malingering, or refusal to take part or continue the study. Out of 413 patients, 282 (68.1%) were females. The flowchart of the patients is presented in Figure 1.

Being exposed to environmental trigger factors (374, 90.6%) was the top prevalent variable of the demographics; and post-traumatic stress disorder (PTSD) (19, 4.5%) was the least prevalent accompanying condition with CDH. Demographics and psychiatric comorbidities related to the intensity of CDH are demonstrated in Table 1.

**Table1:** Demographics and Psychiatric Comorbidities Associated With the Intensity of Chronic Daily Headache

**Domain**	**Variable**	**Frequency (%)**	**P value**
**Demographics**	Environmental triggers	374 (90.6%)	0.2
	Gender (female)	282 (68.3%)	0.02
	Marriage rate	312 (75.5%)	0.23
	Low education (≤ High-school diploma)	248 (60.0%)	0.11
	Chronic primary headache	236 (57.1%)	0.84
	Unhealthy nutrition	214 (51.8%)	0.53
	Headache in family	232 (56.2%)	0.003
	Being an immigrant	203 (49.15%)	0.34
	Low physical activity	201 (48.7%)	0.25
**Psychiatric comorbidities**	Being stressed	372 (90.1%)	0.29
	Anxiety disorders	362 (87.0%)	0.09
	Sleep problems	323 (78.2%)	0.19
	Depressive disorders	261 (63.2%)	0.16
	Obsessive-compulsive disorder	162 (39.2%)	0.93

**Figure 2:**
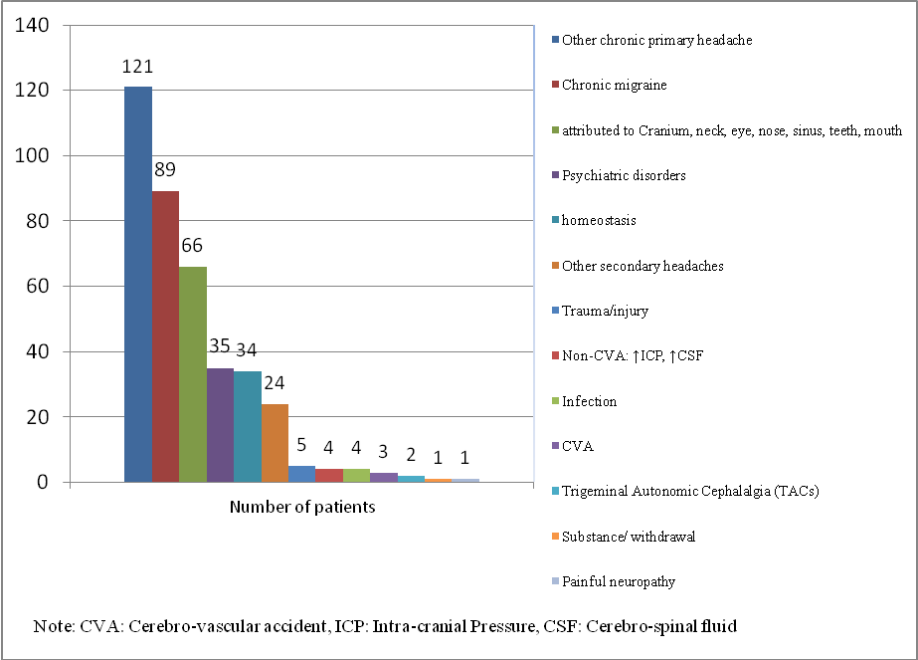
The Chronic Daily Headache Sub-Types Based on ICHD-3 Beta Categories

***Type of Chronic Daily Headache (CDH*** ***(***

Primary headache was reported in 236 cases (57.1%); and most of them, (121 and 51.3%) met the criteria for “other primary headache disorders” according to the ICHD-3 beta version. Chronic migraine (CM), chronic tension-type headache (CTTH), and chronic cluster headache / trigeminal autonomic cephalalgias (TACs) were documented in 89 (37.7%), 24 (10.2%), and 2 (1.7%) patients, respectively, as chronic primary headache types. Headache attributed to disorder of the cranium, neck, eyes, ears, nose, sinuses, teeth, mouth, or other facial or cervical structure was the most frequent type of chronic secondary headache, with 66 patients (37.3%) (Figure2).

***Environmental Trigger Factors***

Those patients living with a person suffering from chronic pain stated more VAS score in comparison to those who did not experience the same condition (mean rank 222.06 vs. 187.69, p<0.003). Most of the patients (368; 89.1%) lived in Tehran or Karaj (the 2 highly populated cities) for at least past 5 years. Among the patients with chronic primary headache (236, 56.9%), 215 (91.10%) were living in the 2 crowded cities compared to 21 (8.89%) patients who lived in other cities. Although the proportion of chronic primary headache /chronic secondary headache was high in the large cities compared to the others in the country (215/21≈10.24 vs. 153/24≈6.34), the difference in the intensity of CDH was not statistically significant (Fisher’s exact test, p<0.09). 

***Intensity of Headache and History of Previous Psychotherapy Correlated to CDH***

Patients were asked to rate their head pain with VAS. The intensity of headache was stated as moderate to severe (mean score = 7.1+/- 1.9, range 2-10), while the most frequent reported VAS score was 8. Patients with previous history of psychotherapy reported significantly higher VAS score compared to those who did not receive the modality (7.49 +/- 1.7 vs. 6.87 +/- 1.9, p<0.001). In addition, females' VAS score was significantly higher than that of the males (mean rank 216.05 vs. 187.51, p<0.02).

## Discussion

Our study had high number of female patients with CDH. Prominence of females in the study has been previously reported. For example, Tellez-Zenteno et al. (2005) reported that 80% of the migraineurs were females (21). The percentage of females with CDH varies from 2% to 4% in adults (22). Females also reported higher VAS score than males. Possible reasons could be related to hormonal fluctuation in women during premenstruation, menstruation, pregnancy, or lactation (23, 24). In addition, the physiological differences and social role perceptions of women may be the reason for their reporting their pain more than men in the same conditions (25).

Intensity of headache was significantly different in patients who were living with a person who suffered from headache in their families. Besides the heritability of migraine and probable genetic risk factors (26), it may be due to learned chronic illness behavior. Whitehead et al. (1982) have reported that patients with irritable bowel syndrome (mainly psychological based (27)) were more likely to show social learning behaviors than patients with peptic ulcer, who did not differ from the general population (28). A review by Turk et al. (1987) demonstrated that patients with chronic pain are in mutual interaction with their families and that their response to the perpetuating pain may affect their health behavior (29). 

Considering the intensity of CDH, 149 (36.0%) of the patients in our study had previously received psychotherapy for their headache. This indicates better psychiatric health services in IR Iran compared to Turkey, which has been reported to be about 25% (30). This also rationalizes the psychological and psychiatric evaluation of patients with CDH during their medical visits (3). 

Patients with CDH reported notable psychiatric problems such as being stressed (90.1%), having anxiety (87%), sleep problems (78.2%), and depression (63.2%) at least in the past 5 years; however, none of the comorbidities was significantly correlated with the intensity of CDH (Table 1). The role of anxiety and stress in CTTH and/or in CM has been well- documented before (31-33). In addition to sleep problems, these account for at least 80% of the triggers in migraine patients with aura (34). Furthermore, stressful life events followed by intense emotions were reported by Abma et al. (2012) as the most frequent triggers in all types of primary headache (35).

Environmental factors were almost as frequent as stress (90.6%) in the present study, but did not differ significantly with the intensity of CDH. The factors have been reported to be one of the important triggers of primary headache in other studies (19, 36 and 37). Markedly and Pathak (2008) reported that about 90% of the patients with headache mentioned that their headache was triggered by noise pollution (38). In our study, those who lived in crowded cities of Tehran or Karaj had significantly more primary headache than patients with secondary headache. Similar results have been reported by Chen (2006), mentioning that environmental factors cause primary headache more than the secondary one (39). 

Traffic related pollution in Iran, especially in big cities such as Tehran, has increased in the past few years due to several reasons such as the use of locally manufactured vehicle fuels, increase of private and/or public vehicles, and imposed sanctions (40). One- thirds of the total days in Tehran were unhealthy, especially for patients with chronic illnesses (12). This situation, as reported by Stansfeld (2000) may reactivate previous or worsen the current psychiatric conditions (41).

In our study, primary headache cases were referred more than the secondary type to the university hospital-based headache clinic, which was similar to the findings by Aaseth (2008) in the general population (42). Although tension-type headache is the most prevalent type of headache in the general population (43, 44), especially in rural areas (45), migraine was observed more than other types of primary headache in the study according to Karakurum Goksel (2014) who reported 64.1% and 18.2% for CM and CTTH, respectively (46). Other researchers such as Katsarava (2009), Yoon (2012), and Gual (2011) have reported similar results (30, 47 and 48). It may come out of a crucial need for the medication usage among the patients with CM headache (49, 50) because the patients prefer to consult with physicians who prescribe modern medications (psychiatrists, neurologists, etc) instead of those who do not such as psychologists, traditional medicine practitioners, and chiropractors (51). 

Of our CDH patients, 29% were labeled as having other primary headache disorders (Figure 2) according to the ICHD-3 beta classification. It was more than what was common; however, it was because some of these patients did not fully meet the criteria for a single sub-type of headache or did not follow their routine medical visits. The other reason was the preference of physicians to categorize the condition of their patients with a single diagnosis (52). 

The high prevalence of patients with lower education in our survey (considering Diploma as the cutoff point), is similar to that of Baherami (2012), who reported that 747 (74.7%) out of 1000 headache cases were female and only 19.7% had university degrees education in the country (53). Moreover, Atasoy (2005) reported that patients with low educational level have poor control of their headache (54).

Another factor in our study was the low physical activity of the patients (48% of cases). Most of CDH sufferers avoided exercising due to fear of worsening their headache (55). It is remarkable that with the exception of the active phase of the headache, all the patients could benefit from scheduled physical activity, preferably under the supervision of an expert physician/therapist (56) to reduce frequency of their pain attacks and improve their cognitive status (57). A systematic review by Baillie (2013) reported positive outcome of headache after aerobic exercise (58). Due to the high levels of smoke, noise, and air pollution in the large and crowded cities (59), the patients should be advised to consider status of air and noise pollution before they go out for exercise. 

Lack of compliance to the recommended nutritional regimen was found in more than half of our patients. This problem is modifiable and depends on the nutritional habits, sedentary lifestyle, and dramatically growing use of fast foods (60, 61). 

## Limitations

The present study was carried out at one referral center, so the generalization of results should be considered with caution. The patients were not matched with respect to social class or income level. We could not include the 135 patients who were excluded from the study as they were unreliable and showed some signs of malingering. As most of the variables did not abide by normal distribution, we applied non-parametric statistical tests.‎

## Conclusion

Patients with CDH suffer from complicated and problematic conditions in their daily life. Coexistence of psychiatric problems and environmental triggers in highly populated cities may double the effect of the burden of headache precipitation or reactivate psychiatric comorbidities. This condition calls for joint psychological- psychiatric interventions to modify the patients’ lifestyle as well as manage environmental triggers. We recommend more studies targeting various aspects of this serious health problem.
